# The current situation and future directions for the study on time-to-pregnancy: a scoping review

**DOI:** 10.1186/s12978-022-01450-6

**Published:** 2022-06-25

**Authors:** Xiang Hong, Jiechen Yin, Wei Wang, Fanqi Zhao, Hong Yu, Bei Wang

**Affiliations:** 1 Key Laboratory of Environmental Medicine and Engineering of Ministry of Education, Department of Epidemiology and Health Statistics, School of Public Health, Southeast University, No.87 Dingjiaqiao Rd, Gulou District, Nanjing, Jiangsu China; 2 Department of Obstetrics and Gynecology, Zhong Da Hospital, School of Medicine, Southeast University, Nanjing, Jiangsu China

**Keywords:** Time-to-pregnancy, Fecundability, Fertility, Influencing factors

## Abstract

**Introduction:**

As problems associated with infertility and population aging increase, there is a growing interest in the factors that cause a decline in human fertility. Time-to-pregnancy (TTP) is a good indicator with which to reflect human fecundability. Here, we present a comprehensive overview of this topic.

**Methods:**

Relevant qualitative and quantitative studies were identified by searching the Web of science and PubMed electronic databases. We included all literature, written in English, from inception to the 10th April 2021 providing the focus was on TTP. We conducted a narrative synthesis using thematic analysis.

**Results:**

Traditional TTP-related study protocols include prospective and retrospective cohorts that provide a wealth of data to reveal potential influences on TTP. Thus far, a variety of factors have been shown to be associated with TTP in couples preparing for pregnancy, including basic demographic characteristics, menstrual status, chronic disease status, environmental endocrine disruptor exposure, and lifestyles. However, there are inevitable epidemiological bias in the existing studies, including recall bias, selection bias and measurement bias. Some methodological advances have brought new opportunities to TTP research, which make it possible to develop precision interventions for population fertility. Future TTP studies should take advantage of artificial intelligence, machine learning, and high-throughput sequencing technologies, and apply medical big data to fully consider and avoid possible bias in the design.

**Conclusion:**

There are many opportunities and future challenges for TTP related studies which would provide a scientific basis for the “precise health management” of the population preparing for pregnancy.

## Plain language summary

As the problems of infertility and population aging increase, there is a growing interest in the factors that cause a decline in human fertility. Time-to-pregnancy (TTP) is a good indicator with which to reflect human fecundability, and a longer TTP is known to reflect a reduction in fertility. Many original studies, with different designs, have used TTP to explore the factors that might influence fertility, including basic demographic characteristics, chronic disease status, environmental endocrine disruptor exposure, and lifestyles. However, much of the existing evidence is inconsistent and limited by various types of bias. This review provides a synopsis of recent TTP studies, and highlights new opportunities and future challenges.

## Introduction

With the further aggravation of population aging, the problem of decreasing birth rate has created wide concern in the international community [1]. A reduced desire to procreate is a complex problem, which can be impacted by social, economic, and psychological factors [2]. Meanwhile, impaired human fecundability can ultimately lead to many couples failing to achieve pregnancy [3]. Existing data show that the global prevalence of infertility ranges from 8 to 12%, but in some areas, can approach 30%. [4] Even with existing clinical diagnostic techniques, almost 40% of all infertile cases are attributed to unexplained factors [5]. Therefore, it is important to investigate the risk factors for human fecundability.

The current diagnostic criteria define infertility as the failure to achieve a successful pregnancy after 12 months or more of regular, unprotected sexual intercourse [6]. This definition means that there are no good physiological indicators to diagnose infertility. Currently, the most commonly used fertility-related biomarkers include male semen quality, female sex hormone levels, and the evaluation of ovarian function [7]. Regrettably, it is difficult to identify a critical value for these indices to direct clinical practice. The general level of sperm quality in men is declining; [8] therefore, it is difficult to define normal sperm quality. Previous research showed that anti-Müllerian hormone was cannot be used to evaluate female fecundability even though it is directly related to ovarian function [9]. In particular, if semen quality and sex hormone levels are tested for every pregnancy-planning couple, there will be a substantial cost. However, this seems questionable without a clear strategy for intervention. In contrast, the presence of some abnormal indicators might induce mental stress; this may have potential impact on pregnancy outcome.

Thus, the evaluation of fecundability remains a significant problem that restricts the development of research in this field. Time-to-pregnancy (TTP), which not only focuses on pregnancy outcome, but also the length of time it takes to achieve pregnancy, appears to represent an important conceptual breakthrough. This index, can be used to identify specific influencing factors for fecundability as this would provide more credible evidence than infertility based case–control studies. Several popular studies have focused on TTP, including the Home Observation of Peri-conceptional Exposures study, [10] the Longitudinal Investigation of Fertility and the Environment study, [11] the Danish National Birth Cohort study, [12] the Norwegian Mother and Child Cohort, [13], and the Singapore Preconception Study of Long-Term Maternal and Child Outcomes study [14]. However, due to various limitations, TTP studies have not led to any significant advancements over the last 20 years. In this study, we summarize the present status, opportunities and challenges for TTP-related research and provide new ideas for solving these limitations based on an epidemiological perspective.

## Methods

In order to review the existing literature relating to TTP, we performed a detailed literature search and developed a selective strategy.

### Research question

There are three main research questions: What are the main types of study design focused on TTP? What are the known influencing factors of TTP from current population study data? What is the next step for TTP related studies?

### Relevant types of evidence

A search for qualitative and quantitative studies was conducted using Web of science and PubMed electronic databases. We included all literature, written in English, from inception to the 10th April 2021 providing the focus was on TTP. “Time to pregnancy” was used as a key search term. Some studies were supplemented using the snowball method of consulting the bibliographies of the articles identified by electronic literature searches.

### Study selection

We included both qualitative reviews and original studies focused on human TTP. The exclusion criteria were as follows: (1) animal studies; (2) original studies that did not use TTP to evaluate human fecundability; (3) duplicated studies. Initially, 2128 articles were identified by the two databases. 862 articles were then excluded as they were repetitive, and 358 studies were excluded because their focus was not appropriate. Consequently, 908 studies were available for qualitative assessment.

### Collation of data, summaries, and reporting results

Narrative descriptions of the evidence were written for each theme. All authors reviewed the descriptions to improve the clarity and relevance and avoid redundancy.

## Main text

### Research designs of TTP-related studies

The traditional designs of TTP related studies were prospective and retrospective cohorts. In a prospective cohort, couples who are planning to attempt for pregnancy are included and the baseline exposure factors can be measured. Then, TTP can be recorded accurately through follow-up. Meanwhile, life-style changes during the pregnancy attempting period can be recorded; this data can make up for the bias from the single measurement of baseline characteristics [15]. In addition, a prospective design can also improve the accuracy of the evaluation outcome, especially biochemical pregnancy and early abortion, which would be missed if a retrospective investigation was performed. However, some limitations should be noted: (1) the cost of cohort maintenance is huge and sample sizes can be limited [16]; (2) frequent follow-up might potentially put pressure on couples, especially for couples with a longer TTP, although infrequent follow-up might miss some outcome information, and (3) follow-up is a potential method of intervention for couples which may involve unpredictable confounding effects for the association between the factors that we focus upon and fecundability.

A retrospective cohort design would always include pregnant women and then ask them to recall the TTP and some exposure information [17]. This design has a lower cost and is simple and practicable. However, there are obvious limitations. First, recall bias is inevitable. A previous study carried out a TTP survey on women for 10 years after they gave birth; just 19% of women managed to recall information accurately [18]. However, another study [19] found that recalled TTP showed good agreement with prospective TTP. Second, selection bias; all of the participants are pregnant or have a history of pregnancy; the couples failing to achieve pregnancy are difficult to recruit. Thus, some couples with risk factor exposure may be missed, thus underestimating the effect of this factor [20]. Third, the lifestyle changes during the pregnancy attempting period cannot be recorded.

Beyond these designs, some studies have used a “nested case–control” design and defined the concept of “subfertility” to compare situations between subfertile and normal women. Usually, women who fail to achieve a successful pregnancy after 6 months are defined as subfertile [21, 22]. The major problem with this design is outcome measurement bias; the subjective division of outcome would bring unexpected bias for association estimation.

Over recent decades, real world data (RWD) are increasingly being taken into account and applied by the academic community [23]. This approach always uses big data, including medicine information systems, insurance systems, and cause-of-death surveillance systems, to present a true medical situation. There have been few international studies on TTP using RWD. In China, some studies used data from the National Free Pre-pregnancy Health Check-up Project; this data was collected conventionally to explore the risk factors of TTP, including body mass index (BMI) [24], blood pressure status [15] and the vaginal microenvironment [25]. RWD-based studies present some advantages, including low implementation costs, huge sample sizes, and reduced choice bias (all the participants are from a natural population, including infertile and fertile couples). Moreover, the application of certain statistical analysis techniques, such as propensity score matching and post-randomization, the strength of evidence from RWD can achieve similar levels to that of randomized controlled trials [26]. However, there are some challenges associated with RWD studies. Due to the lack of targeted design, some information related to human fecundability are not collected in some databases, such as semen quality and the frequency of intercourse. Furthermore, RWD are always collected from different medical centers; consequently, data quality can vary. Finally, the large sample size makes small associations statistically significant; this may increase the rate of false positives [27].

### The factors that influence TTP

Initially, researchers focused on the adverse effects of occupational exposure factors on TTP, and then quickly spread to many other fields, including endocrine disruption, the menstruation status, chronic disease, and exposure to drugs.

#### Basic demographic characteristics and TTP

Although basic demographic characteristics are immutable factors for couples, they are important for establishing a prediction model. Age is the most important and definitive factor for TTP; researchers continue to define an optimal age of fertility. Some studies reported that the association between female age and fertility forms an inverted u-shape and that the optimal range may be 20–35 years-of-age; however, this remains controversial [28, 29]. By contrast, other researchers have reported that female fertility declines with the age of 20 years, without an optimal range [30, 31]. It has yet to be determined if there is an optimal age for males [28, 31] and Scheike et al. [29] reported that male fecundability declines with age.

The associations between occupational exposures and TTP have been widely reported. Greenhouse workers, especially those exposed to pesticides, are known to exhibit reduced fecundability [32–34]. Exposure to lead has been proven to be an independent risk factor for fecundability [34]. Compared with administrative workers, domestic workers seem to have a significantly lower per-cycle probability of conception [35]. In addition, the negative association between shift work and fecundability is still controversial [36, 37].

A women’s history of pregnancy is thought to be associated with TTP. The American LIFE cohort found that women experiencing pregnancy loss had a longer TTP [38]. Wildenschild et al. found that the reduction in fecundability was greater for women with repeated miscarriage [39]. However, interestingly, women with a history of induced abortion be associated with a slight increase in fertility [40]. The main reason for induced abortion is unintended pregnancy; thus to some extent, this might reflect the higher fecundability of such women. However, repeated induced abortion may cause damage to the uterine environment, thus increasing the risk of secondary infertility [41].

In a large cohort, Zhang et al. [42] found that a later onset of menarche, a longer menstrual cycle length, and both a shorter (< 4 days) and longer (> 5 days) duration of bleeding, were associated with a lower fecundability and TTP in rural Chinese women. These findings are generally consistent with other studies [43, 44]. In addition, irregular menstruation appears to be a risk factor for fecundability [43]. Female menstruation status is a reflection of the functionality of the hypothalamus-pituitary-ovary axis, and needs to be investigated further.

#### Chronic disease status and TTP

Excessive weight and obesity are topics that are often implicated in medical scenarios. Gesink et al. [45] found that obese women showed an 18% reduction of fecundability; many similar studies support these findings [24, 46, 47]. However, whether excessive weight or obesity exerts an adverse effect on male fecundability has yet to be determined. This is because existing standards for excessive weight and obesity in males appear to be unsuitable for fecundability evaluation [24]. In reality, ethnic differences may also impact the association between BMI and fecundability [48, 49]. In addition, being underweight also appears to be a risk factor, although there is less evidence for this association [24, 50].

Metabolic syndrome, including hypertension and diabetes is an important factor affecting human fecundability. Zhao et al. found that women with impaired fasting glucose levels had an 18% reduction in fecundability [51]. Furthermore, Whitworth et al. reported that type 1 or type 2 diabetes could damage female fertility, even among those with a normal menstrual cycle [13]. In addition, our previous study found that couples with hypertension (a systolic blood pressure of more than 140 mm Hg or a diastolic blood pressure of more than 90 mm Hg) have a lower fecundability [15]. However, these associations were not statistically significant in other studies [52].

#### Exposure to environmental endocrine disruptors (EDCs) and TTP

EDCs are a class of chemicals that can interfere with normal endocrine systems or technology and can be commonly found in food, daily necessities, and cosmetics. Many animal studies have focused on EDCs and reproductive toxicity [53, 54]. However, direct evidence from TTP studies remains insufficient. Recently, some EDCs, such as polychlorinated biphenyls, [55, 56] bisphenol A [57], and phthalates [58], have been reported to be associated with a longer TTP. However, findings were not consistent when compared between different studies; therefore, this research requires further confirmation [59].

#### Lifestyles and TTP

The adverse effect of smoking on fertility is well accepted [60]. Sapra et al. [61] analyzed cohort data and showed that smoking reduces the fecundability by almost 50% in both males and females. In addition, passive smoking is becoming an accepted concern. Radin et al. explored the association between passive smoking and fecundability, but did not find significant association [62]. Recently, Harlow et al. focused on the E-cigarettes and found that current e-cigarette use was associated with slightly reduced fecundability [63]. E-cigarettes, electronic games, and short video addiction, need to be investigated further with regards to fecundability.

Although drinking alcohol is considered unhealthy behavior, the effect on fecundability is controversial. Fan et al. [64] performed a meta-analysis that included 19 original articles and found that alcohol consumption by females was associated with reduced fecundability, but did not provide a threshold value for daily intake. The specific mechanism underlying the damaging effects of alcohol on female fecundability remains unclear. One hypothesis is that women with drinking habits are likely to have high levels of estrogen, thus inhibiting follicle-stimulating hormone; this would exert impact on follicular maturation and ovulation [65]. For males, the concept of moderate alcohol consumption remains controversial. Jensen et al. found that males who drank a small amount of alcohol each week did not show any adverse effects in terms of sperm concentration and the concentration of free testosterone [66]. Although semen quality and alcohol intake have been widely studied, it has not been determined whether lower semen quality reduces fertility [67, 68]. Bonde et al. found that semen volume and motility were of limited value when predicting pregnancy [69]. Thus, we can put forward the hypothesis that there is a threshold effect for male alcohol consumption on fecundability and that minor damage to sperm quality may not affect male fertility. In contrast, Florack et al. found a weak positive correlation between male fecundability and moderate drinking, [70] which was probably due to the confounding effect of the frequency of sexual behavior.

In addition, the benefits of physical activity in women attempting pregnancy have been widely accepted, especially for walking among those with a higher BMI [71]. The non-linear relationship between physical activity and time-to-pregnancy has already been investigated [72], and generated new evidence for health guidance pre-pregnancy. Coffee and caffeine intake were regarded as a potential impact factor for fecundability, but with inconsistent evidence. [73, 74]

#### Other aspects

With regards to TTP, more and more potential influential factors have been recognized with the development of research methods. Dietary factors, including pre-pregnancy fast food, fruit intake, [75] and seafood intake habits [76]. Disease factors include inflammatory bowel diseases, [77] asthma, [78], and uterine fibroids [79]. Drug use factors include psychotropic medication, [80] marijuana, [81] oral contraceptives, [82], and pain-relievers [83]. Mental factors include pre-pregnancy perceived stress [84] and depression [80]. Gaining an understanding of these emerging factors will contribute to the comprehensive construction of a TTP-related influencing factors network to facilitate the accurate prediction of infertility.

### Opportunities and challenges for TTP-related studies

With the development of biostatistics and genomics technology, medical research has gradually focused on artificial intelligence, machine learning, high-throughput sequencing, and other fields; these tools provide new opportunities for revealing the secrets of life science. At present, TTP-related studies are mostly based on traditional epidemiological designs, although there is significant scope for innovative research, as detailed below.

1. Genomics technology and TTP. A recent study found that variation in the *HLA-F* gene is associated with TTP [85]. genome wide association studies provide the best chance of studying the association between single nucleotide polymorphisms (SNPs) and fecundability, although little progress has been made in this area. SNPs will help to construct genetic risk scores for fecundability which will help to implement targeted intervention strategies.
2. Microbial multi-omics and TTP. The human body normally carries a huge abundance of microorganism, especially in the intestinal tract, oral cavity, vagina, and skin; these areas play important roles in human health. Studies have indicated that the gut and vaginal microbiome are potential factors for infertility [86, 87]. However, no association studies between the human microbiome and TTP have been reported thus far. When considering microbial multi-omics study, it is evident that there are many levels of research, including the metagenomics, macrotranscriptomics, metaproteomics, and metabonomics; all of which will help us to understand the mechanisms of how the human microbiome could impact fertility.
3. Environmental exposure omics and TTP. Although the TTP index was first used to explore the impact of occupational and environmental exposure, many questions remain unanswered. When considering novel compound materials and the combined effects of multiple exposures, it is necessary to study the exposure omics of environmental pollutants. Some compounds with reproductive toxicity should be verified in TTP cohort studies.
4. Real world data and TTP. As mentioned earlier, TTP cohort designs are costly to organize and maintain. With the further standardization and sharing of medical big data, the integration of health insurance data, pre-conception health screening data, census data, and birth/death registration data, will hopefully provide a richer data resource for TTP studies in the future. Real world data will help to implement post hoc randomization to address the bias of some key unmeasured confounding factors.
5. Mathematical algorithm development and TTP. At present, the most common analytical tools used in traditional epidemiology in the analysis of TTP-related studies are logistic and Cox regression models. However, these generalized linear models cannot fully meet the needs of future massive data analysis. With the development of machine learning technology, the mining of high-dimensional data is no longer a problem. In the future, it is expected that machine learning algorithms will be used to build predictive models for the assessment of TTP; these will eventually facilitate the primary prevention of clinical infertility.

Of course, a good study design is what guarantees the scientific validity of a particular study. TTP study designs, in addition to the limitations mentioned in earlier, also feature other possible biases that need to be considered in future. First, temporal bias. As production technology advances, the work environment may change over time; when a study is carried out over time, it needs to take into account that there are differences in the risk of individuals being exposed to the risk of exposure factors in different time periods. Second, family planning policy bias. Especially in China, the family planning policy has undergone several reforms and the effects of these reforms on fertility intention should be considered when we conduct TTP studies. Third, planned pregnancy bias. Study subjects are always couples actively preparing for pregnancy; those with unintended pregnancies would not be included; inevitably, this is the group that may have generally high levels of fertility [20]. Of course, it is possible that this bias could be avoided if medical big data resources were well utilized. Fourth, medical intervention bias. Lifestyle, health status, and other indicators of couples in the pregnancy preparation process should be considered a dynamic process. However, most of the current studies focus only on the results of one preconception test. Finally, the effects of unhealthy workers. In contrast to the common “healthy worker effect”, TTP studies will show an increased duration and probability of remaining on the job for workers who have been preparing for pregnancy for a long time and are not pregnant. Once pregnancy is confirmed, many workers will leave their jobs, thus resulting in the false association of work exposure time as a risk factor for infertility.

## Conclusion

With the prominence of infertility problems, it will be imperative to conduct fertility and TTP-related studies to promote the primary prevention of infertility. Traditional TTP research protocols include prospective and retrospective cohorts that provide a wealth of data to reveal potential influences on TTP. Thus far, a variety of factors have been shown to be associated with fertility in couples preparing for pregnancy, including basic demographic characteristics, menstrual status, chronic disease status, environmental endocrine disruptor exposure, and lifestyle. However, there is still a great deal of scope to achieve “precision interventions” for population fertility. Future TTP studies should take advantage of artificial intelligence, machine learning, and high-throughput sequencing technologies, and take advantage of medical big data to fully consider and avoid possible bias in study design, so as to provide a scientific basis for the “precise health management” of the population preparing for pregnancy.

## Data Availability

The datasets used and/or analyzed during the current study are available from the corresponding author on reasonable request.

## Abbreviations

TTP: Time to pregnancy
RWD: Real world data
EDCs: Environmental endocrine disruptors
SNP: Single nucleotide polymorphisms
